# Mycoplasma and cancer: in search of the link

**DOI:** 10.18632/oncotarget.264

**Published:** 2011-04-19

**Authors:** Melissa B. Rogers

**Affiliations:** Biochemistry & Molecular Biology, UMDNJ - NJ Medical School, Newark, New Jersey

Identifying links between specific infectious agents and cancer greatly impacts the prevention of these cancers. Furthermore, understanding the role of cellular factors that mediate the effect of infectious agents may reveal additional cancer control strategies. A potential association between cancers and infection with mycoplasma, the smallest self-replicating prokaryote, has been suspected since the 1960's (reviewed in [1]). However, early evidence was weakened by the difficult culture conditions demanded by these fastidious microbes. More recent studies have used techniques such as PCR, immunohistochemistry, and serum antibody status. These improved detection methods have shown that healthy individuals are often colonized without obvious clinical effects [2, 3]. Later studies continue to support an association between various species of mycoplasma and human cancers (e.g., [4-8]).

This study by Barykova *et al*. [7] is the latest of several that indicate a strong link between mycoplasma species and prostate cancer [8, 9]. Barykova *et al*. used real time PCR to demonstrate that *Mycoplasma hominis* levels in biopsies from patients with high-grade prostatic intraepithelial neoplasia (HGPIN) or prostate cancer (PCa) were triple that of patients with benign prostatic hyperplasia (BPH, p = 0.002). Other species commonly found in the urogenital tract, *M. genitalium* and *Ureaplasma urealitycum*, were detected at a lower frequency. Most significantly, no mycoplasma species were detected in control samples from lesion free men. Culturing confirmed the presence of *M. hominis* and *U. urealitycum* in prostate tissue (*M. genitalium* was not cultured). Finally, antibodies against *M. hominis* protein p120 were detected in the serum of HGPIN or PCa patients twice as frequently as in patients with BPH. The Barykova et al. study is corroborated by another study that determined that PCa patients were more likely to bear antibodies against *U. urealitycum* [8]. Finally, *M. genitalium* and *M. hyorhinis* infection caused the malignant transformation of benign human prostate (BPH-1) cells *in vitro* [9].

Laboratory studies strongly support the ability of mycoplasma to cause or promote oncogenic transformation. Several different species have been proven to transform rodent and human lines of diverse lineages *in vitro* [9-12]. Many plausible mechanisms would explain this pro-cancer effect: the induction of genetic instability [11, 13, 14], alterations in metabolism [2, 3, 15], and dramatic changes in the expression of many genes. Specific genes include known tumor suppressors and oncogenes [16, 17] and many potent cell signals, such as pro-inflammatory cytokines and other growth factors [15, 18-21]. Infected tumor cells *or* adjacent infected cells may synthesize cytokines and growth factors that promote the growth of nascent tumor cells. Increased growth rate, along with increased mutation rate, would facilitate the transformation of infected cells.

We were the first to demonstrate that mycoplasma oncogenically transformed human lung cells [12]. We also discovered that mycoplasma species rank among the best known inducers of bone morphogenetic protein (BMP) 2 [12]. This is particularly relevant to lung cancer, because BMP2 RNA and protein levels are abnormally elevated in lung tumors [22-24]. BMP2 activates pro-oncogenic pathways (*e.g.*, PI3K/mTOR; Smad1,5/Id-1) and promotes lung tumor growth in mice [25-27]. The BMP2 antagonist, noggin, reduces mixed metastatic lung cancer lesions in bone [28] and the growth of transformed lung cells in monolayer and in soft agar [12]. Finally, high BMP2 levels are associated with poor patient survival [29].

At the least, mycoplasma infection is a potential biomarker for prostate malignancy. At best, the prostate cancer/mycoplasma link suggests that more aggressive antibiotic treatment of mycoplasma-infected men may be beneficial. Although smoking is the most important cause of lung cancer, other factors contribute to the development of lung cancer [30]. Proof that mycoplasma also influences lung cancer would facilitate the prevention and/or treatment of lung cancer. In addition, understanding the signals such as BMP2 that mediate the effect of mycoplasma may reveal other therapeutic options for a disease that is the leading cause of cancer deaths in the United States.
